# Maturation in Serum Thyroid Function Parameters Over Childhood and Puberty: Results of a Longitudinal Study

**DOI:** 10.1210/jc.2016-3605

**Published:** 2017-05-01

**Authors:** Peter N. Taylor, Adrian Sayers, Onyebuchi Okosieme, Gautam Das, Mohd S. Draman, Arshiya Tabasum, Hussam Abusahmin, Mohammad Rahman, Kirsty Stevenson, Alix Groom, Kate Northstone, Wolf Woltersdorf, Andrew Taylor, Susan Ring, John H. Lazarus, John W. Gregory, Aled Rees, Nicholas Timpson, Colin M. Dayan

**Affiliations:** 1 Thyroid Research Group, Systems Immunity Research Institute, Cardiff University School of Medicine, Cardiff CF14 4XN, United Kingdom; 2 Department of Social and Community Medicine, University of Bristol, Bristol BS8 2BN, United Kingdom; 3 Musculoskeletal Research Unit, University of Bristol, Learning and Research, Southmead Hospital, Westbury on Trym, Bristol BS10 5NB, United Kingdom; 4 Endocrinology and Diabetes Department, Prince Charles Hospital, Cwm Taf University Health Board, Merthyr Tydfil CF47 9DT, United Kingdom; 5 Endocrinology and Diabetes Department, University Hospital of Wales, Cardiff CF14 4XN, United Kingdom; 6 Department of Biochemistry, Bristol Royal Infirmary University Hospitals Bristol NHS Foundation Trust, Bristol BS2 8HW, United Kingdom; 7 MRC Integrative Epidemiology Unit, University of Bristol, Bristol BS8 2BN, United Kingdom; 8 Facharzt für Laboratoriumsmedizin Geschäftsleiter MVZ Labor, Dr. Reising-Ackermann and Kollegen Strümpellstrasse, 40 04289 Leipzig, Germany; 9 Department of Biochemistry, Royal United Hospital, Bath BA1 3NG, United Kingdom; 10 Neuroscience and Mental Health Research, Cardiff University School of Medicine, Cardiff CF24 4HQ, United Kingdom

## Abstract

**Context:**

Serum thyroid hormone levels differ between children and adults, but have not been studied longitudinally through childhood.

**Objective:**

To assess changes in thyroid-stimulating hormone (TSH) and thyroid hormone levels over childhood and their interrelationships.

**Design:**

Cohort study.

**Setting:**

The Avon Longitudinal Study of Parents and Children, a population-based birth cohort.

**Participants:**

A total of 4442 children who had thyroid function measured at age 7, and 1263 children who had thyroid function measured at age 15. Eight hundred eighty-four children had measurements at both ages.

**Main Outcome Measures:**

Reference ranges for TSH, free tri-iodothyronine (FT_3_), free thyroxine (FT_4_), their longitudinal stability, and interrelationships.

**Results:**

Children at age 7 years had a higher FT_3_ [6.17 pmol/L, standard deviation (SD) 0.62] than children at age 15 (5.83 pmol/L, SD 0.74); *P* < 0.0001 with 23.2% of children at age 7 having FT_3_ above the adult reference range. Higher FT_3_ levels at age 7 in boys (*P* = 0.0001) and girls (*P* = 0.04) were associated with attainment of a more advanced pubertal stage at age 13. TSH was positively associated with FT_3_ at age 7 and age 15 even after adjusting for confounders. In contrast, TSH was negatively associated with FT_4_.

**Conclusions:**

There are substantial changes in TSH and thyroid hormone levels over childhood, in particular for FT_3_, which appear to relate to pubertal readiness. Our data provide increased insight into the evolution of the pituitary–thyroid axis over childhood and may have implications for determining optimal ranges for thyroid hormone replacement in children.

## Teaser

We studied thyroid function in children at ages 7 and 15 years. We identified FT3 levels were substantially higher at age 7, and higher levels of FT3 were associated with more advanced puberty.

Thyroid hormones play an important role in developmental processes, including growth, maintenance of metabolic balance, and cell development (1). Even minor variation in thyroid hormone status within the normal population reference range is associated with important phenotypic consequences (2). The complex inverse relationship between thyroid-stimulating hormone (TSH) and free thyroxine (FT_4_) renders TSH the more sensitive marker of overall thyroid status (3). Free tri-iodothyronine (FT_3_) is the active thyroid hormone, although serum levels only indirectly reflect overall thyroid status because a substantial proportion of intracellular FT_3_ is produced from conversion of intracellular FT_4_ by deiodinases (4, 5). However, there is some evidence that T_3_ may have a more important role than previously assumed in both the assessment and therapy of thyroid disease in younger children (6).

Thyroid hormone levels are largely genetically determined (7), with similar effects from genetic variation observed in children and adults (8). Although it is well established in adults that there is narrow intraindividual variation in thyroid hormone parameters compared with interindividual variation (9), increased variance and ranges in thyroid hormone levels have been observed throughout childhood, and adult reference intervals may not be universally applicable to children (10–12). Previous cross-sectional studies have indicated that FT_3_ substantially falls and FT_4_ rises from age 4 (13–15), but there have been no longitudinal studies to confirm these observations. Furthermore, from genetic analyses we have recently identified that higher body mass index and adiposity appear to causally increase FT_3_, but not TSH or FT_4_ levels (16); therefore, the longitudinal stability of thyroid hormones over childhood, and FT_3_ in particular, remains unclear.

In this report, we studied TSH and thyroid hormone levels at ages 7 and 15 in a large population birth cohort. We assessed age and sex reference ranges in 4442 healthy children at age 7 and 1253 children at age 15 (884 children had thyroid function measured at both time points). We also explored the longitudinal variability of TSH and thyroid hormone levels using linear mixed models by sex, pubertal status, and body mass index (BMI) and also assessed the relationship between TSH and thyroid hormone at different time points over childhood.

## Methods

### Participants

Avon Longitudinal Study of Parents and Children (ALSPAC) is a prospective birth cohort that enrolled >13,000 pregnant women in the former County of Avon, UK, with an expected delivery date between April 1991 and December 1992 (17, 18) (see www.alspac.bris.ac.uk). Children were regularly brought back to focus clinics where data were collected and phenotypic measurements and blood samples were taken. The study website contains details of all the data that are available through a fully searchable database: www.bris.ac.uk/alspac/researchers/data-access/data-dictionary/. Ethical approval for the study was obtained from the ALSPAC Ethics and Law Committee and the Local Research Ethics Committees. There were no children on levothyroxine or antithyroid medications in the study dataset.

### Laboratory measures

TSH, FT_3_, and FT_4_ were measured during 2010–2011 on remaining frozen stored serum samples taken from the focus at age 7 years (median age 89 months) and focus at age 15 clinics (median age 184 months). Samples were analyzed using chemiluminescent emission utilizing a photomultiplier on cobas e601 (Roche Diagnostics, Mannheim, Germany). A total of 4442 samples was available for full thyroid function testing at age 7 years, and 1253 were available at age 15 years. A total of 884 children samples was available and processed at both ages 7 and 15. Reference ranges for adults are TSH, 0.27 to 4.2 mU/L; FT_3_, 3.9 to 6.7 pmol/L; and FT_4_, 12 to 22 pmol/L. It has been previously demonstrated that TSH and FT_4_ can be analyzed reliably in samples stored for up to 23 years (19). The intra-assay precision coefficients of variance for TSH, FT_3_, and FT_4_ were <3.1%, <4%, and <4%, respectively. The interassay precision coefficients of variance were <7.3%, <6%, and <7%, respectively.

### Phenotypic measures

Standing height was measured using a wall-mounted Harpenden stadiometer (Holtain, Crymych, UK). BMI was calculated as weight (in kilograms) divided by height (in meters) squared. Pubertal status was self-assessed using a Tanner stage questionnaire at age 13.5 years (pubic hair domain), range 13.1 to 14.4 years.

### Statistical analysis

Implausible TSH and thyroid hormone levels [>4 standard deviation (SD) from the mean for the sex- and age-specific category] were considered as outliers and were recoded to missing. TSH was log_e_ transformed to an approximately normal distribution. Descriptive statistics are presented as geometric means, SD, median, and 95th centiles.

A linear mixed model with random intercepts and random slopes was used to assess the trends of TSH and thyroid hormone parameters over childhood (20). An unstructured variance–covariance matrix was assumed. We analyzed the baseline values at age 7, the variability at baseline, the longitudinal trend (slope) between ages 7 and 15, and the variability in the slope. Analyses were performed with gender interactions and gender X puberty interactions. Model simplification was undertaken using likelihood ratio tests. Additional analysis was undertaken adjusting for BMI, as this may be associated with pubertal development and FT_3_ in particular or on the causal pathway between thyroid status and pubertal development.

We then explored the relationship between TSH and thyroid hormone levels at ages 7 and 15. Here thyroid function was standardized, and therefore results are presented as per SD change in the outcome. Analyses were initially performed adjusted for age at thyroid measurements and gender (model 1). Three further models controlling for key potential confounders were undertaken; model 2 also adjusted for thyroid hormone parameters, model 3 also adjusted for measures of social class and early life environment including parents’ home ownership, maternal age at birth of child, maternal highest educational qualification, maternal smoking in pregnancy, family adversity index, and parents and home score. Likelihood ratio tests were used to identify whether there was any evidence of interaction by sex on the relationship between thyroid hormone parameters and TSH.

## Results

### Study population and baseline characteristics

**Figure 1.**
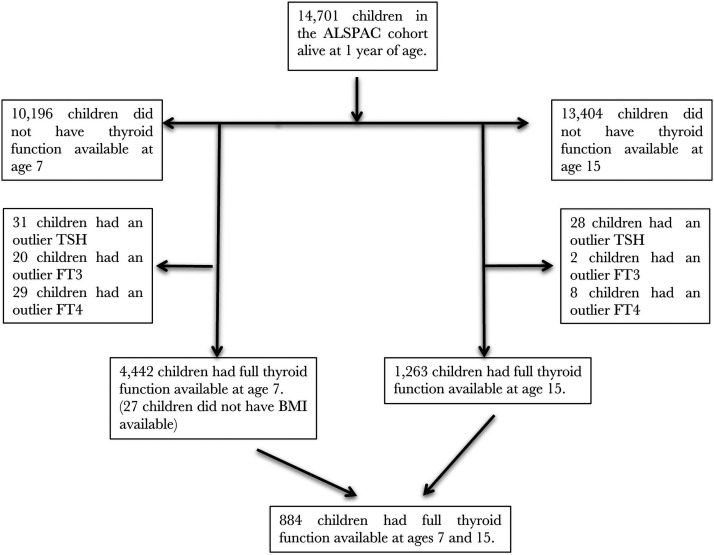
Study participants.

The derivation of study participant numbers is shown in Fig. 1. A total of 80 children at age 7 (1.8%) and 38 children at age 15 (2.9%) met the outlier exclusion. Children in our final analysis dataset were more likely to have several higher markers of affluence and fewer early life events than the remainder of the ALSPAC cohort (Supplemental Table 1).

### Serum thyroid hormone levels in children at ages 7 and 15

**Figure 2.**
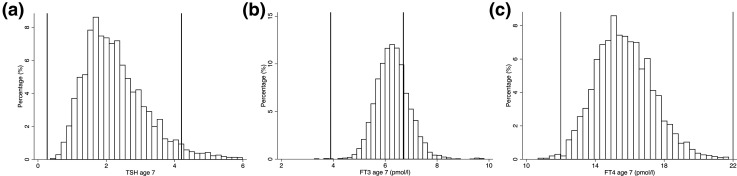
(a) Histogram of TSH levels at age 7 (vertical lines refer to adult reference range). (b) Histogram of FT_3_ levels at age 7 (vertical lines refer to adult reference range). (c) Histogram of FT_4_ levels at age 7 (vertical lines refer to adult reference range).

**Figure 3.**
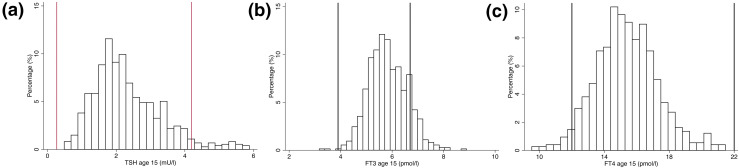
(a) Histogram of TSH levels at age 15 (vertical lines refer to adult reference range). (b) Histogram of FT_3_ levels at age 15 (vertical lines refer to adult reference range). (c) Histogram of FT_4_ levels at age 15 (vertical lines refer to adult reference range).

At age 7 years, the mean and 95% reference range values for TSH, FT_3_, and FT_4_ were 2.26 (0.93 to 4.48) mU/L, 6.29 (5.13 to 7.59) pmol/L, and 15.7 (12.7 to 19.3) pmol/L, respectively (Table 1). A total of 23.2% of children at age 7 years had a FT_3_ above the adult reference range, with only 3.65% of children having a TSH and 0.2% of children having FT_4_ values above the adult reference range (Fig. 2; Table 1). At age 15 years, the mean and 95% reference range values for TSH, FT_3_, and FT_4_ were 2.43 (0.91 to 5.05) mU/L, 5.83 (4.45 to 7.35) pmol/L, and 15.5 (11.9 to 20.3) pmol/L, respectively (Fig. 3; Table 1), with a marked reduction in children having FT_3_ above the adult reference range to 12.2%, which was mainly in girls (Table 1). Analysis of just the 884 children who had thyroid function at both ages 7 and 15 revealed similar results (Supplemental Table 2). There was a modest correlation between TSH levels between ages 7 and 15 (Pearson’s correlation coefficient = 0.35), which was similar for FT_4_ (Pearson’s correlation coefficient = 0.33), although a much weaker correlation was observed for FT_3_ (Pearson’s correlation coefficient = 0.10). Bland–Altman plots revealed no evidence of heteroskedasticity for TSH, FT_3_, and FT_4_ (Supplemental Fig. 1).

**Table 1.** Reference Range for Thyroid Hormone Parameters Age 7 and Age 15

		All					Males					Females				
	Age (Y)	N	Mean	(2.5%–97.5%)	% Above ARR	% Below ARR	N	Mean	(2.5%–97.5%)	% Above ARR	% Below ARR	N	Mean	(2.5%–97.5%)	% Above ARR	% Below ARR
TSH (mU/L)		4442	2.26	0.93–4.48	3.65	0	2323	2.32	0.97–4.50	3.57	0	2119	2.20	0.88–4.45	3.73	0
FT3 (pmol/L)	7	4442	6.29	5.13–7.59	23.2	0.09	2323	6.23	5.07–7.56	19.8	0.17	2119	6.35	5.16–7.59	26.9	0
FT4 (pmol/L)		4422	15.7	12.7–19.3	0.20	0.70	2323	15.6	12.7–19.0	0.17	0.73	2119	15.9	12.85–19.55	0.24	0.66
TSH (mU/L)		1263	2.43	0.91–5.05	6.33	0	644	2.51	0.91–5.17	7.92	0	619	2.34	0.87–5.00	4.68	0
FT3 (pmol/L)	15	1263	5.83	4.45–7.35	12.2	0.55	644	6.16	4.84–7.6	20.7	0	619	5.48	4.23–6.91	3.39	1.13
FT4 (pmol/L)		1263	15.5	11.9–20.3	0.79	2.69	644	15.5	11.8–20.2	0.62	2.95	619	15.5	12.0–20.6	0.97	2.42

Abbreviation: ARR, adult reference range.

### Linear mixed models analysis in children with thyroid function at age 7 and age 15

TSH levels rose between ages 7 and 15 years, whereas both FT_3_ and FT_4_ levels fell. Strong negative correlations were observed in the models for TSH FT_3_ and FT_4_, indicating that those with higher levels at age 7 years were more likely to have more substantial lowering of levels at age 15, and those with lower levels at age 7 were likely to have smaller reductions at age 15, *i.e.*, a convergence of biomarkers (Table 2). Every 2 years between ages 7 and 15 years, TSH levels increased by 0.03 mU/L [95% confidence interval (CI) 0.02, 0.05], *P* < 0.001. Boys had a higher baseline TSH than girls at age 7 years by 0.11 mU/L (95% CI 0.06, 0.17), *P* < 0.001. There was no difference in mean gain between boys and girls between ages 7 and 15 years, B = 0.0001 (95% CI −0.001, 0.001), *P* = 0.83, and no difference in variability at baseline −0.04 (95% CI −0.10, 0.03), *P* = 0.29, or in the variability of the slope B = 5.73 × 10^−06^ (95% CI −0.0002 and 0.0003), *P* = 0.65 (Table 2).

**Table 2.** Overall Linear Mixed Models for TSH FT_3_ and FT_4_

Parameter	Group	Measure	Factor	Coefficient	95% CI	*P* Value
TSH (mU/L)	All	Main effects	Age 7 years	2.27	(2.24, 2.3)	<0.001
			Slope	0.0013	(0.0007, 0.002)	<0.001
		Variability	SD@ Age 7 years	1.62	(1.56, 1.67)	
			SD Slope	0.14	(0.13, 0.14)	
			Correlation (int, slope)	−0.87	(−0.89, −0.86)	
FT3 (pmol/L)	All	Main effects	Age 7 years	6.29	(6.27, 6.31)	<0.001
			Slope	−0.005	(−0.005, −0.004)	<0.001
		Variability	SD@ Age 7 years	1.28	(1.24, 1.32)	
			SDSlope	0.12	(0.11, 0.12)	
			Correlation(int, slope)	−0.92	(−0.93, −0.91)	
FT4 (pmol/L)	All	Main effects	Age 7 years	15.7	(15.7, 15.8)	<0.001
			Slope	−0.002	(−0.03, −0.0005)	0.005
		Variability	SD@ Age 7	3.03	(2.92, 3.14)	
			SDSlope	0.27	(0.26, 0.28)	
			Correlation (int, slope)	−0.86	(−0.88, −0.84)	
TSH	Boys	Main effects	Age 7	2.32	2.28, 2.36	<0.001
			Slope	0.001	0.0005, 0.002	0.002
		Variability	SD@ Age 7	0.9	0.87, 0.92	
			SDSlope	0.01	0.01, 0.01	
			Correlation (int, slope)	−0.46	−0.52, −0.39	
FT3	Boys	Main effects	Age 7	6.23	6.2, 6.25	<0.001
			Slope	−0.0005	−0.001, 0.000009	0.09
		Variability	SD@ Age 7	0.63	0.61, 0.64	
			SDSlope	0.009	0.008, 0.009	
			Correlation (int, slope)	−0.58	−0.63, −0.53	
FT4	Boys	Main effects	Age 7	15.5	15.4, 15.6	<0.001
			Slope	0.0003	−0.001, 0.002	0.72
		Variability	SD@ Age 7	1.63	1.58, 1.68	
			SDSlope	0.022	0.021, 0.023	
			Correlation (int, slope)	−0.41	−0.48, −0.34	
TSH	Girls	Main effects	Age 7	2.21	2.17, 2.24	
			Slope	0.001	0.0004, 0.002	<0.001
		Variability	SD@ Age 7	0.92	0.89, 0.95	
			SDSlope	0.01	0.01, 0.01	
			Correlation (int, slope)	−0.52	−0.58, −0.46	
FT3	Girls	Main effects	Age 7	6.36	6.33, 6.38	<0.001
			Slope	−0.009	−0.01, −0.08	<0.001
		Variability	SD@ Age 7	0.61	0.59, 0.63	
			SDSlope	0.009	0.008, 0.009	
			Correlation (int, slope)	−0.61	−0.66, −0.56	
FT4	Girls	Main effects	Age 7	15.9	15.8, 16	<0.001
			Slope	−0.004	−0.006, −0.002	<0.001
		Variability	SD@ Age 7	1.74	1.69, 1.8	
			SDSlope	0.022	0.21, 0.22	
			Correlation (int, slope)	−0.42	−0.49, −0.35	

For FT_3_, every 2 years between the ages of 7 and 15 years, FT_3_ levels fell 0.12 pmol/L (95% CI −0.13, −0.10). Girls had a higher baseline FT_3_ level than boys by 0.13 pmol/L (95% CI 0.09, 0.17), *P* < 0.001. However, boys had a reduced decline in FT_3_ than girls, B = 0.008 (95% CI 0.007, 0.009), *P* < 0.001. There was no substantial difference by sex in variability at baseline B = 0.02 (95% CI −0.01, 0.05), *P* = 0.29, or in variability in slope B = 7.85 × 10^−06^ (95% CI −5.18 × 10^−06^, 2.01 × 10^−05^), *P* = 0.24 (Table 2). Every 2 years, FT_4_ levels fell 0.04 pmol/L (95% CI −0.07, −0.01), *P* = 0.005. Girls had a higher baseline FT_4_ level than boys by 0.38 pmol/L (95% CI 0.28, 0.48), *P* < 0.001, and also had more variability at baseline at age 7 years, B = 0.38 (95% CI 0.14, 0.62), *P* = 0.002, although there was no difference in variability in slope B = 4.47 × 10^−05^ (95% CI 4.47 × 10^−05^, 0.001), *P* = 0.33 (Table 2). Adjusting the analysis for BMI revealed similar results, although it markedly attenuated the slope for TSH (Supplemental Table 3).

### Relationship between pubertal status at age 13 and TSH and thyroid hormone parameters at ages 7 and 15

A total of 2702 children also had pubertal status self-assessed at age 13 years as well as having thyroid function measured. As expected, girls had a higher Tanner score than boys 3.63 (95% CI 3.58, 3.69) vs 2.96 (95% CI 2.89, 3.02), *P* < 0.0001. Pubertal status at age 13 years was not associated with TSH levels at age 7 in boys (*P* = 0.89) or girls (*P* = 0.31). No difference in TSH slope by pubertal status was observed in boys (*P* = 0.82) or girls (*P* = 0.82). Pubertal status at age 13 years was also not associated with FT_4_ levels at age 7 years in boys (*P* = 0.32) or girls (*P* = 0.52). By contrast, FT_3_ levels at age 7 years were higher in both boys (*P* = 0.0001) and girls (*P* = 0.04) with more advanced puberty at age 13 years (Table 3). More advanced pubertal status at age 13 years was, however, associated with a negative FT_3_ slope unlike children at an earlier pubertal status at age 13, which had a positive FT_3_ slope, in both boys and girls (*P* ≤ 0.001). Similarly, there was no evidence of any difference in the variability of baseline values or gradients of slopes by pubertal status in either boys or girls for either FT_3_ or FT_4_. Although BMI at age 7 was also associated with Tanner stage at age 13, B = 0.08 (95% CI 0.06, 0.09), *P* < 0.001, and FT_3_, B = 0.04 (95% CI 0.03, 0.05), *P* < 0.001, adjusting for BMI at age 7 had no substantial effect on the relationship between FT_3_ and Tanner stage. Analysis of the association between FT_3_ and Tanner stage when adjusted for sex was B = 0.12 (95% CI 0.07, 0.12), *P* < 0.001; adding BMI to the model had a minimal impact on effect estimates, B = 0.10 (95% CI 0.05, 0.15), *P* < 0.001. Furthermore, adjustment for BMI in the linear mixed models performed by pubertal status revealed very similar results to our original analysis (Supplemental Table 4).

**Table 3.** Linear Mixed Models for TSH FT_3_ and FT_4_ by Pubertal Status at Age 13 Years

				P1			P2			P3		
Parameter	Group	Measure	Factor	Coefficient	95% CI	*P* Value	Coefficient	95% CI	*P* Value	Coefficient	95% CI	*P* Value
TSH	Boys	Main effects	Age 7	2.37	2.28, 2.46	<0.001	2.4	2.3, 2.51	<0.001	2.38	2.29, 2.46	<0.001
			Slope	0.002	0, 0.004	0.05	0.001	−0.001, 0.003	0.19	0.001	−0.001, 0.002	0.26
		Variability	SD@ Age 7	0.92	0.85, 0.98		0.94	0.86, 1.01		0.94	0.88, 1.01	
			SDSlope	0.01	0.01, 0.01		0.01	0.01, 0.01		0.01	0.01, 0.01	
			Correlation (int, slope)	−0.47	−0.59, −0.34		−0.41	−0.57, −0.26		−0.46	−0.58, −0.33	
T3	Boys	Main effects	Age 7	6.14	6.08, 6.2	<0.001	6.18	6.11, 6.25	<0.001	6.32	6.26, 6.38	<0.001
			Slope	0.002	0.0003, 0.003	0.01	0	−0.001, 0.001	0.99	−0.003	−0.004, −0.002	<0.001
		Variability	SD@ Age 7	0.63	0.59, 0.68		0.59	0.54, 0.64		0.63	0.58, 0.67	
			SDSlope	0.008	0.007, 0.009		0.01	0.008, 0.01		0.009	0.008, 0.01	
			Correlation (int, slope)	−0.53	−0.64, −0.41		−0.66	−0.77, −0.56		−0.62	−0.71, −0.52	
T4	Boys	Main effects	Age 7	15.6	15.4, 15.7	<0.001	15.6	15.5, 15.8	<0.001	15.5	15.3, 15.6	<0.001
			Slope	−0.004	−0.007, −0.001	0.02	−0.004	−0.008, −0.001	0.02	0.005	0.002, 0.009	0.001
		Variability	SD@ Age 7	1.58	1.47, 1.69		1.6	1.46, 1.73		1.66	1.54, 1.77	
			SDSlope	0.02	0.02, 0.03		0.02	0.02, 0.03		0.02	0.02, 0.03	
			Correlation (int, slope)	−0.5	−0.62, −0.38		−0.5	−0.64, −0.36		−0.33	−0.47, −0.19	
TSH	Girls	Main effects	Age 7	2.16	2.04, 2.28	<0.001	2.28	2.17, 2.39	<0.001	2.2	2.13, 2.27	<0.001
			Slope	0.0004	−0.001, 0.002	0.71	0.001	−0.001, 0.003	0.21	0.001	−0.003, 0.002	0.15
		Variability	SD@ Age 7	0.86	0.76, 0.94		0.95	0.87, 1.02		0.92	0.88, 0.97	
			SDSlope	0.01	0.01, 0.01		0.01	0.01, 0.01		0.01	0.01, 0.01	
			Correlation (int, slope)	−0.6	−0.74, −0.45		−0.57	−0.7, −0.45		−0.52	−0.61, −0.44	
T3	Girls	Main effects	Age 7	6.27	6.19, 6.36	<0.001	6.27	6.2, 6.34	<0.001	6.37	6.32, 6.41	<0.001
			Slope	−0.007	−0.009, −0.005	<0.001	−0.007	−0.008, −0.006	<0.001	−0.01	−0.01, −0.01	<0.001
		Variability	SD@ Age 7	0.6	0.54, 0.66		0.63	0.57, 0.68		0.62	0.59, 0.65	
			SDSlope	0.008	0.007, 0.01		0.008	0.006, 0.009		0.009	0.008, 0.01	
			Correlation (int, slope)	−0.77	−0.86, −0.68		−0.57	−0.7, −0.44		−0.6	−0.67, −0.52	
T4	Girls	Main effects	Age 7	15.9	15.6, 16.2	<0.001	15.8	15.6, 16	<0.001	15.9	15.8, 16	<0.001
			Slope	−0.004	−0.008, 0.0003	0.07	−0.002	−0.006, 0.002	0.43	−0.004	−0.006, −0.001	0.005
		Variability	SD@ Age 7	1.88	1.68, 2.06		1.79	1.64, 1.93		1.77	1.68, 1.86	
			SDSlope	0.02	0.02, 0.02		0.02	0.02, 0.03		0.02	0.02, 0.03	
			Correlation (int, slope)	−0.42	−0.61, −0.23		−0.43	−0.58, −0.27		−0.43	−0.53, −0.33	

### Relationship between TSH and serum thyroid hormone levels in children at ages 7 and 15 years

At age 7 years, TSH was weakly positively associated with FT_3_ after adjusting for age, sex, FT_4_, and markers of social class and early life environment, B standardized (std) = 0.03 (95% CI 0.001, 0.06), *P* = 0.05, whereas TSH was clearly negatively associated with FT_4_, B (std) = −0.07 (95% CI −0.10, −0.04), *P* = 3.49 × 10^−05^ (Supplemental Table 5). A similar pattern was also observed at age 15 years even after adjusting for pubertal status, with TSH positively associated with FT_3_, B (std) = 0.07 (95% CI 0.02, 0.13), *P* = 0.01, and negatively associated with FT_4_, B (std) = −0.13 (95% CI −0.19, −0.07), *P* = 5.16 × 10^−06^ (Supplemental Table 5). FT_3_ and FT_4_ were positively associated with each other at age 7 years, B (std) = 0.27 (95% CI 0.24, 0.30), *P* = 1.12 × 10^−14^, and also at age 15 years, B = 0.19 (95% CI 0.12, 0.26), *P* = 4.23 × 10^−07^. Seemingly unrelated regression identified that the positive impact of TSH on FT_3_ was greater at age 15 years than at age 7 years (*P* = 0.001), but no difference was observed with FT_4_ (*P* = 0.84).

## Discussion

Our results from a longitudinal analysis of a large population birth cohort demonstrate that there are substantial changes in the pituitary–thyroid axis over childhood. In particular, FT_3_ changes much more over childhood than either TSH or FT_4_. Levels of FT_3_ at age 7 are high compared with adult values, with almost 25% of children at age 7 years having a FT_3_ level above the adult reference range. Although there is a substantial fall in FT_3_ levels between age 7 years and age 15 years, 10% are still above the adult reference range.

There was a very strong negative correlation between hormone levels between ages 7 and 15, indicating that the substantial variability observed in early childhood is reduced through puberty, with hormone levels converging to near adult reference values. Overall, our data suggest that there may be higher conversion of FT_4_ to FT_3_ in younger children than adults. Our observation that boys maintain a higher FT_3_ for longer than girls is also noteworthy, and may have substantial importance in observed sex differences in bone development (21) and other phenotypes (2).

The reason that children have higher FT_3_ levels at age 7 years is unclear but may be due to factors external to the pituitary–thyroid axis, such as fat mass and pubertal development (16). In the current study, we noted that children that reached puberty earlier (as indicated by more advanced self-reported pubertal stage at age 13 years) had higher FT_3_ values at age 7 years and a negative FT_3_ slope between ages 7 and 15 years, whereas those with less advanced puberty had a positive FT_3_ slope between ages 7 and 15 years. Using both serum thyroid function and then genetic data to perform Mendelian Randomization, we have recently reported that BMI and fat mass in children are positively and causally related to FT_3_ (16). Although the effect of FT_3_ on puberty is interestingly largely independent of BMI, it is, however, still possible that FT_3_ is an indicator of nutritional state and hence pubertal readiness in early childhood in a manner similar to leptin. Alternatively, the observed changes may represent changes in the thyroid gland in preparation for puberty, or be a consequence of changes in other endocrine factors such as growth hormone, as growth hormone therapy has been linked to marginally increased FT_3_ and decreased FT_4_ levels (22).

We have also identified a difference in the relationship between TSH and the two thyroid hormones FT_3_ and FT_4_ in childhood, with higher TSH being associated with higher FT_3_, whereas an inverse association was identified with FT_4_. The positive association between TSH and FT_3_ in childhood has been highlighted recently in children with borderline thyroid status (23). This observation provides insight into childhood TSH-FT_4_ and TSH-FT_3_ relationships that are relevant to our understanding of both thyroid physiology and the laboratory diagnosis of thyroid disease. It is interesting to speculate the life course of FT_3_ levels given it is well established that FT_3_ in particular declines in the elderly (24); the pattern of FT_3_ through life may therefore be a fall over childhood (25), then plateauing throughout adult life, before falling again in older age.

We believe our findings are also clinically relevant, given the striking differences observed in early childhood thyroid hormone levels from adult-derived reference ranges. If age- and sex-appropriate reference ranges are not used, there may be substantial overdiagnosis of subclinical thyroid disease in children. In addition, our finding that children have substantially higher FT_3_ levels than adults may have implications for thyroid hormone replacement in children. Individuals on levothyroxine have a higher FT_4_ and a lower FT_3_ than euthyroid individuals despite having similar TSH levels (26–28). Children on levothyroxine might therefore have inadequate FT_3_ levels for optimal timing of puberty and other developmental processes. It is noteworthy that hypothyroidism diagnosed in prepubertal years can cause a delay of puberty (29). It is also possible that the relative lack of FT_3_ in these children may potentially be one of the reasons that optimal IQ levels are not reached in children with congenital hypothyroidism despite adequate levothyroxine therapy (30). Taken together, there remains a pressing need for further study of central and peripheral determinants of thyroid function as well as determinants of intracellular thyroid status in children.

Strengths of our dataset include the use of a large population birth cohort with detailed phenotypic data available and paired thyroid function at two age points, which allows more robust analysis than previous studies of cross-sectional samples. The nature of the cohort means it is unlikely that interfering medications or heterophilic antibodies have influenced results. Furthermore, our use of liner mixed models has allowed us to determine the change of TSH and thyroid hormone levels between ages 7 and 15, while simultaneously adjusting for an individual’s baseline hormone levels, allowing us to investigate how variability reduces as children progress into adulthood. Limitations of our study include a higher social class bias in our dataset and lack of generalizability to ethnic minorities, as 98% of all samples analyzed were in individuals of Caucasian descent. A weakness is that paired samples were also not all performed on the same assay run. Furthermore, all individuals were from a small region of the UK that has been shown to be borderline iodine deficient (31). Our findings require replication in individuals from other ethnic groups and using different thyroid hormone assays from an area of iodine sufficiency.

In conclusion, our results demonstrate that thyroid hormone levels change substantially during childhood and adolescence. This is particularly the case with FT_3_, which is substantially higher in younger children. FT_3_ levels also appear to influence the onset of puberty; further studies into the pituitary–thyroid axis in normal childhood populations are therefore needed to define the role of higher FT_3_ levels in childhood more precisely.
