# Supplementary material for: Maturation in Serum Thyroid Function Parameters Over Childhood and Puberty: Results of a Longitudinal Study
Source: J Clin Endocrinol Metab. 2017 May 1;102(7):2508–15. doi: 10.1210/jc.2016-3605 (PMC5505201; doi:10.1210/jc.2016-3605)
Supplement: Supplementary file 1 [file jc.2016-3605.sd1.docx]

**Supplementary Online Material**

**Text**

Supplementary Text 1 Details of Confounders used in analysis

**Tables**

Supplementary Table 1 Comparison of the study cohort (N=4,422) to remainder of the ALSPAC cohort.

Supplementary Table 2 Reference-range for thyroid hormone parameters age 7 and age 15 in the 884 individuals with TSH FT3 and FT4 at both time points

Supplementary Table 3 Linear mixed models for TSH FT_3_ and FT_4_ adjusted by BMI tertile at age 7

Supplementary Table 4 Linear mixed models for TSH FT_3_ and FT_4_ by pubertal status at age 13 years adjusted by BMI tertile at age 7

Supplementary Table 5 Relationship between TSH and other thyroid hormone parameters (standardized) at age 7 and age 15

**Figures**

Supplementary Figure 1A Bland Altman Plot for TSH at age 7 and age 15

Supplementary Figure 1B Bland Altman Plot for FT_3_ at age 7 and age 15

Supplementary Figure 1C Bland Altman Plot for FT_4_ at age 7 and age 15

**Supplementary Text 1**

Analyses were adjusted for several key confounders

**Child derived**

Sex of child

Age of child at assessments (months)

Thyroid hormone parameters

**Mother and Family**

Age of mother at birth of child

Parity of mother at birth of child (1/1-4/more than 4)

Maternal smoking during pregnancy (none/some)

Educational status of mother (low=no qualifications, certificate of secondary education, or vocational/medium=O level/high=A level or degree)

Housing status (owned or mortgaged/privately rented/council rented)

Family adversity index (see below)

Home score (1-4/4-8/9-12 -see below)

- **Family Adversity Index**

A measure of hardship during pregnancy and early life

Sum of (1 point given for each applicable item):

**Demographics**

- Mother younger than 20 years at first pregnancy

**Housing**

- Housing inadequacy (crowding and periods of homelessness)

- Basic living conditions (no availability of hot water,

no indoor toilet, bath or shower, or no kitchen)

- Major defects in housing or infestation

**Education**

- No educational qualifications (mother or father)

**Financial status**

- Financial difficulties

**Relationship with partner**

- Single status

- Low affection and aggression

- Physical/emotional cruelty

- No social support

- Family

- Family size >4 children

- Caregiving problems (on social services risk register,

child in care/not with natural mother)

**Social network**

- No emotional support
- No practical/financial support

**Maternal emotional status**

- Depression, anxiety or suicide attempts

- Substance abuse

**Drugs or alcohol use**

- Crime

- In trouble with police

- Actual convictions

- **Home observation for measurement of environment (HOME) score**

Assessed at six months postpartum and a measure of the emotional and cognitive environment

Variable derived from responses to:

- Child has cuddly toys

- Child has push/pull toys

- Child has co-ordination toys

- Number of books child has of their own

- Mother teaches child

- Mother talks to child when working

**Supplementary Table 1** Comparison of the study cohort (N=4,422) to remainder of the ALSPAC cohort.

| **Variable** | **Study Cohort** | **Remaining ALSPAC cohort*** | **p value** |
| --- | --- | --- | --- |
| **Child Sex (% Male)** | 52.3 | 50.9 | 0.14 |
| **Family Adversity Index Mean (SD)** | 4.67 (4.42) | 3.88 (3.94) | <0.001 |
| **Home Score**  0-4 (%)  5-8 (%)  9-12 (%) | 3.39  24.0  72.6 | 4.64  24.0  72.5 | 0.003 |
| **Housing Status**  Owned/mortgaged (%)  Privately rented (%)  Council rented/other (%) | 82.9  13.3  3.8 | 69.1  25.1  5.8 | <0.001 |
| **Maternal age at birth of child (years) Mean (SD)** | 29.1 (4.55) | 27.5 (5.06) | <0.001 |
| **Maternal highest educational status**  Low **(%)**  Middle (%)  High (%) | 20.9  35.1  44.0 | 34.4  34.3  31.3 | <0.001 |
| **Maternal smoking in pregnancy**  None (%)  Some (%) | 82.1  17.9 | 72.2  27.8 | <0.001 |
| **Parity**  0 - 1 (%)  2 – 4 (%)  > 5 (%) | 81.1  18.7  0.2 | 79.2  20.2  0.7 | <0.001 |

Calculated using the Wald test

*Remaining ALSPAC cohort defined as women who enrolled in the core ALSPAC sample with children surviving to 1 year (N= 14,701) p = strength of evidence against the null hypothesis of no difference in characteristics between study population and remainder of the ALSPAC cohort

S**upplementary Table 2** Reference-range for thyroid hormone parameters age 7 and age 15 in the 884 individuals with TSH FT3 and FT4 at both time points

|  | Age (years) | All | | | | | Males | | |  |  | Females | | |  |  |
| --- | --- | --- | --- | --- | --- | --- | --- | --- | --- | --- | --- | --- | --- | --- | --- | --- |
|  |  | N | Mean | (2.5-97.5%) | %  above  ARR | %  below  ARR | N | Mean | (2.5-97.5%) | %  above  ARR | %  below  ARR | N | Mean | (2.5-97.5%) | %  above  RR | %  below  RR |
| TSH (mU/l) |  | 884 | 2.42 | 0.97 - 4.53 | 3.85 | 0 | 441 | 2.22 | 0.99- 4.83 | 4.76 | 0 | 443 | 2.22 | 0.95 – 4.34 | 2.93 | 0 |
| FT3 (pmol/l) | 7 | 884 | 6.11 | 5.05 – 7.55 | 18.6 | 0 | 441 | 6.23 | 4.99 – 7.59 | 16.1 | 0 | 443 | 6.24 | 5.10- 7.54 | 21.0 | 0 |
| FT4 (pmol/l) |  | 884 | 15.2 | 12.6 – 19.0 | 0.11 | 0.68 | 441 | 15.6 | 12.6 – 18.5 | 0 | 0.68 | 443 | 15.6 | 12.7 – 19.2 | 0.23 | 0.68 |
|  |  |  |  |  |  |  |  |  |  |  |  |  |  |  |  |  |
|  |  |  |  |  |  |  |  |  |  |  |  |  |  |  |  |  |
| TSH (mU/l) |  | 884 | 2.34 | 0.88 – 4.91 | 4.98 | 0 | 441 | 2.44 | 0.89- 5.20 | 6.58 | 0 | 443 | 2.24 | 0.84 – 4.51 | 3.39 | 0 |
| FT3 (pmol/l) | 15 | 884 | 5.83 | 4.52 – 7.37 | 11.7 | 0.45 | 441 | 6.17 | 4.91 – 7.64 | 20.2 | 0 | 443 | 5.49 | 4.29 – 6.89 | 3.16 | 0.90 |
| FT4 (pmol/l) |  | 884 | 15.4 | 11.9 – 20.1 | 0.34 | 2.71 | 441 | 15.4 | 11.8 – 20.1 | 0.45 | 3.17 | 443 | 15.4 | 12.0 – 19.9 | 0.23 | 2.42 |

**Supplementary Table 3** Overall linear mixed models for TSH FT_3_ and FT_4_ adjusted by BMI tertile at age 7

|  |  |  |  | Coefficient | 95% CI | | P-Value |
| --- | --- | --- | --- | --- | --- | --- | --- |
| TSH  (mU/l) | All | Main effects | Age 7 years | 2.23 | (2.19, | 2.28) | <0.001 |
|  |  |  | Slope | 0.0005 | (-0.0002, | 0.001) | 0.15 |
|  |  | Variability | SD@ Age 7 years | 1.62 | (1.56 | 1.67) |  |
|  |  |  | SD Slope | 0.14 | (0.13, | 0.14) |  |
|  |  |  | Correlation(int,slope) | -0.87 | (-0.89, | -0.86) |  |
| FT3  (pmol/l) | All | Main effects | Age 7 years | 6.23 | 6.20 | 6.26 | <0.001 |
|  |  |  | Slope | -0.004 | -0.005 | 0.004 | <0.001 |
|  |  | Variability | SD@ Age 7 years | 1.27 | (1.22, | 1.31) |  |
|  |  |  | SDSlope | 0.11 | (0.11, | 0.12) |  |
|  |  |  | Correlation(int,slope) | -0.92 | (-0.93, | -0.91) |  |
| FT4  (pmol/l) | All | Main effects | Age 7 years | 15.9 | (15.8, | 16.0) | <0.001 |
|  |  |  | Slope | -0.002 | (-0.003, | -0.0004) | 0.009 |
|  |  | Variability | SD@ Age 7 | 3.03 | (2.92 | 3.14) |  |
|  |  |  | SDSlope | 0.26 | (0.25, | 0.27) |  |
|  |  |  | Correlation(int,slope) | -0.87 | (-0.89, | -0.86) |  |
| TSH | Boys | Main effects | Age 7 | 2.29 | 2.24 | 2.34 | <0.001 |
|  |  |  | Slope | 0.001 | -0.0003 | 0.002 | 0.23 |
|  |  | Variability | SD@ Age 7 | 0.90 | 0.87 | 0.92 |  |
|  |  |  | SDSlope | 0.01 | 0.01 | 0.01 |  |
|  |  |  | Correlation(int,slope) | -0.46 | -0.53 | -0.40 |  |
| FT3 | Boys | Main effects | Age 7 | 6.17 | 6.14 | 6.21 | <0.001 |
|  |  |  | Slope | -0.0004 | -0.001 | 0.0003 | 0.32 |
|  |  | Variability | SD@ Age 7 | 0.62 | 0.61 | 0.64 |  |
|  |  |  | SDSlope | 0.009 | 0.008 | 0.009 |  |
|  |  |  | Correlation(int,slope) | -0.59 | -0.65 | -0.53 |  |
| FT4 | Boys | Main effects | Age 7 | 15.7 | 15.6 | 15.8 | <0.001 |
|  |  |  | Slope | 0.0004 | -0.002 | 0.002 | 0.69 |
|  |  | Variability | SD@ Age 7 | 1.62 | 1.58 | 1.67 |  |
|  |  |  | SDSlope | 0.02 | 0.02 | 0.02 |  |
|  |  |  | Correlation(int,slope) | -0.44 | -0.51 | -0.37 |  |
| TSH | Girls | Main effects | Age 7 | 2.17 | 2.12 | 2.22 | <0.001 |
|  |  |  | Slope | 0.0003 | -0.0006 | 0.001 | 0.45 |
|  |  | Variability | SD@ Age 7 | 0.92 | 0.89 | 0.94 |  |
|  |  |  | SDSlope | 0.01 | 0.01 | 0.01 |  |
|  |  |  | Correlation(int,slope) | -0.56 | -0.62 | -0.50 |  |
| FT3 | Girls | Main effects | Age 7 | 6.29 | 6.26 | 6.32 | <0.001 |
|  |  |  | Slope | -0.009 | -0.01 | -0.08 | <0.001 |
|  |  | Variability | SD@ Age 7 | 0.61 | 0.59 | 0.63 |  |
|  |  |  | SDSlope | 0.009 | 0.008 | 0.009 |  |
|  |  |  | Correlation(int,slope) | -0.63 | -0.68 | -0.57 |  |
| FT4 | Girls | Main effects | Age 7 | 16.1 | 16.0 | 16.2 | <0.001 |
|  |  |  | Slope | -0.004 | -0.006 | -0.002 | <0.001 |
|  |  | Variability | SD@ Age 7 | 1.73 | 1.68 | 1.79 |  |
|  |  |  | SDSlope | 0.02 | 0.02 | 00.2 |  |
|  |  |  | Correlation(int,slope) | -0.49 | -0.56 | -0.42 |  |

**Supplementary Table 4** Linear mixed models for TSH FT_3_ and FT_4_ by pubertal status at age 13 years adjusted by BMI tertile at age 7

|  |  |  |  | P1 | | | | P2 | | | | P3 | | | |
| --- | --- | --- | --- | --- | --- | --- | --- | --- | --- | --- | --- | --- | --- | --- | --- |
|  |  |  |  | Coefficient | 95% CI | | P-Value | Coefficient | 95% CI | | P-Value | Coefficient | 95% CI | | P-Value |
| TSH | Boys | Main effects | Age 7 | 2.37 | 2.27 | 2.47 | <0.001 | 2.40 | 2.28 | 2.52 | <0.001 | 2.38 | 2.28 | 2.49 | <0.001 |
|  |  |  | Slope | 0.001 | -0.001 | 0.003 | 0.28 | 0.001 | -0.002 | 0.003 | 0.65 | 0.001 | -0.002 | 0.003 | 0.61 |
|  |  | Variability | SD@ Age 7 | 0.92 | 0.85 | 0.98 |  | 0.94 | 0.86 | 1.01 |  | 0.95 | 0.88 | 1.01 |  |
|  |  |  | SDSlope | 0.01 | 0.01 | 0.01 |  | 0.01 | 0.01 | 0.01 |  | 0.01 | 0.01 | 0.01 |  |
|  |  |  | Correlation(int,slope) | -0.48 | -0.62 | -0.35 |  | -0.44 | -0.61 | -0.27 |  | -0.43 | -0.58 | -0.29 |  |
| T3 | Boys | Main effects | Age 7 | 6.11 | 6.04 | 6.18 | <0.001 | 6.14 | 6.06 | 6.21 | <0.001 | 6.27 | 6.20 | 6.34 | <0.001 |
|  |  |  | Slope | 0.002 | 0.001 | 0.003 | 0.003 | 0.001 | -0.002 | 0.001 | 0.99 | -0.003 | -0.004 | -0.002 | <0.001 |
|  |  | Variability | SD@ Age 7 | 0.63 | 0.59 | 0.67 |  | 0.59 | 0.54 | 0.64 |  | 0.62 | 0.58 | 0.66 |  |
|  |  |  | SDSlope | 0.008 | 0.007 | 0.009 |  | 0.01 | 0.008 | 0.01 |  | 0.009 | 0.008 | 0.01 |  |
|  |  |  | Correlation(int,slope) | -0.57 | -0.69 | -0.46 |  | -0.68 | -0.78 | -0.58 |  | -0.62 | -0.72 | -0.52 |  |
| T4 | Boys | Main effects | Age 7 | 15.6 | 15.5 | 15.8 | <0.001 | 15.7 | 15.5 | 15.9 | <0.001 | 15.6 | 15.3 | 15.8 | <0.001 |
|  |  |  | Slope | -0.004 | -0.007 | 0.0001 | 0.06 | -0.004 | -0.009 | -0.0001 | 0.05 | 0.006 | 0.002 | 0.009 | 0.001 |
|  |  | Variability | SD@ Age 7 | 1.58 | 1.47 | 1.69 |  | 1.61 | 1.47 | 1.73 |  | 1.65 | 1.53 | 1.75 |  |
|  |  |  | SDSlope | 0.02 | 0.02 | 0.03 |  | 0.02 | 0.02 | 0.03 |  | 0.02 | 0.02 | 0.02 |  |
|  |  |  | Correlation(int,slope) | -0.52 | -0.65 | -0.40 |  | -0.48 | -0.64 | -0.33 |  | -043 | -0.57 | -0.29 |  |
| TSH | Girls | Main effects | Age 7 | 2.16 | 2.03 | 2.29 | <0.001 | 2.27 | 2.15 | 2.39 | <0.001 | 2.20 | 2.11 | 2.28 | <0.001 |
|  |  |  | Slope | 0.0001 | -0.002 | 0.002 | 0.90 | 0.0002 | -0.002 | 0.002 | 0.84 | 0.0003 | -0.002 | 0.002 | 0.65 |
|  |  | Variability | SD@ Age 7 | 0.85 | 0.76 | 0.93 |  | 0.95 | 0.87 | 1.02 |  | 0.92 | 0.87 | 0.97 |  |
|  |  |  | SDSlope | 0.01 | 0.01 | 0.01 |  | 0.01 | 0.01 | 0.01 |  | 0.01 | 0.01 | 0.01 |  |
|  |  |  | Correlation(int,slope) | -0.68 | -0.82 | -0.55 |  | -0.61 | -0.74 | -0.49 |  | -0.55 | -0.64 | -0.46 |  |
| T3 | Girls | Main effects | Age 7 | 6.24 | 6.15 | 6.31 | <0.001 | 6.23 | 6.16 | 6.31 | <0.001 | 6.31 | 6.25 | 6.36 | <0.001 |
|  |  |  | Slope | -0.007 | -0.009 | -0.005 | <0.001 | -0.007 | -0.008 | -0.005 | <0.001 | -0.01 | -0.01 | -0.01 | <0.001 |
|  |  | Variability | SD@ Age 7 | 0.60 | 0.54 | 0.66 |  | 0.62 | 0.58 | 0.68 |  | 0.62 | 0.58 | 0.65 |  |
|  |  |  | SDSlope | 0.008 | 0.007 | 0.01 |  | 0.008 | 0.007 | 0.009 |  | 0.009 | 0.008 | 0.01 |  |
|  |  |  | Correlation(int,slope) | -0.78 | -0.87 | -0.69 |  | -0.62 | -0.75 | -0.50 |  | -0.6 | -0.68 | -0.53 |  |
| T4 | Girls | Main effects | Age 7 | 16.0 | 15.7 | 16.2 | <0.001 | 15.9 | 15.7 | 16.1 | <0.001 | 16.0 | 15.8 | 16.2 | <0.001 |
|  |  |  | Slope | -0.006 | -0.01 | -0.001 | 0.001 | 0.0003 | -0.003 | 0.005 | 0.87 | -0.004 | -0.007 | -0.001 | 0.004 |
|  |  | Variability | SD@ Age 7 | 1.87 | 1.68 | 2.05 |  | 1.78 | 1.63 | 1.92 |  | 1.76 | 1.67 | 1.85 |  |
|  |  |  | SDSlope | 0.02 | 0.02 | 0.02 |  | 0.02 | 0.02 | 0.02 |  | 0.02 | 0.02 | 0.02 |  |
|  |  |  | Correlation(int,slope) | -0.54 | -0.72 | -0.36 |  | -0.52 | -0.67 | -0.36 |  | -0.50 | -0.60 | -0.41 |  |

**Supplementary Table 5** Relationships between TSH and other thyroid hormone parameters (standardized) at age 7 and age 15

| Model | FT3 (pmol/l) | | | FT4 (pmol/l) | | |
| --- | --- | --- | --- | --- | --- | --- |
|  | B (std) | 95%CI | p* | B (std) | 95%CI | p* |
| **Age 7 (N=4,442)** | | | | | | |
| Model 1 | 0.01 | -0.02, 0.04 | 0.56 | -0.07 | -0.10, -0.04 | 1.09x10^-06^ |
| Model 2 | 0.03 | 0.001, 0.06 | 0.05 | -0.08 | -0.10, -0.05 | 1.63x10^-07^ |
| Model 3* | 0.03 | 0.001, 0.06 | 0.05 | -0.07 | -0.10, -0.04 | 3.49x10^-05^ |
| **Age 15 (N =1,263)** | | | | | | |
| Model 1 | 0.06 | 0.01, 0.11 | 0.02 | -0.08 | -0.14, -0.03 | 0.003 |
| Model 2 | 0.08 | 0.03, 0.12 | 0.002 | -0.10 | -0.15, -0.04 | 4.17x10^-05^ |
| Model 3† | 0.08 | 0.03, 0.13 | 0.001 | -0.12 | -0.17, -0.06 | 3.38x10^-06^ |
| Model 4†† | 0.07 | 0.02, 0.13 | 0.01 | -0.13, | -0.19, -0.07 | 5.16x10^-06^ |

* Calculated using the Wald test

B=Beta coefficient

CI = Confidence interval

std = Standardized

p = strength of evidence against the null hypothesis of no association

Model 1 adjusted for age and sex

Model 2 adjusted for Model 1 and other thyroid hormone parameters

Model 3 adjusted for Model 2 and markers of social class and early life environment (home ownership, maternal age at birth of child, maternal highest educational qualification, maternal smoking in pregnancy, family adversity index and parents and home score)

Model 4 adjusted for Model 3 and Tanner stage

*900 individuals with missing data

†88 individuals with missing data

†† 364 individuals with missing data

**Supplementary Figure 1A** Bland Altman Plot for TSH at age 7 and age 15****

**Supplementary Figure 1B** Bland Altman Plot for FT3 at age 7 and age 15

**Supplementary Figure 1C** Bland Altman Plot for FT4 at age 7 and age 15
